# Clinical and Economic Correlates of Pharmacotherapy in Patients with Essential Tremor

**DOI:** 10.5334/tohm.973

**Published:** 2024-12-17

**Authors:** Rajesh Pahwa, Kalea Colletta, Donald Higgins, Bridgette Kanz Schroader, Brian M. Davis, Liana Hennum, Elan D. Louis

**Affiliations:** 1 University of Kansas Medical Center, Kansas City, KS, USA; 2 Department of Neurology, Edward Hines Jr. VA Hospital, Hines, IL, USA; 3 Research and Development, Samuel S. Stratton VA Medical Center, Albany, NY, USA; 4 Cencora (previously AmerisourceBergen/Xcenda), Conshohocken, PA, USA; 5 Merative, Ann Arbor, MI, USA; 6 Former employee, Cala Health, San Mateo, CA, USA; 7 Department of Neurology, University of Texas Southwestern Medical Center, Dallas, TX, USA; 8 Peter O’Donnell Jr. Brain Institute, University of Texas Southwestern Medical Center, Dallas, Texas, USA

**Keywords:** essential tremor, neurologic disorders, clinical burden, economic burden, pharmacotherapy burden, real-world evidence

## Abstract

**Background:**

Essential tremor (ET) is among the most common movement disorders, yet there are few treatment options. Medications have limited efficacy and adverse effects; thus, patients often discontinue pharmacotherapy or take several medications in combination. We evaluated the economic correlates (healthcare resource utilization [HCRU] and costs) and comorbidities among adults with and without ET and among subgroups of patients with ET prescribed 0 to ≥3 ET medications.

**Method:**

This was a retrospective cohort study using claims data from the Merative Market Scan Research Databases (1/1/2017–1/31/2022). Patients were categorized as commercially insured (22–<65 years) or Medicare (≥65 years) and stratified into 3 subgroups: patients with untreated ET, patients with treated ET, and non-ET patients. The index date was the date of first ET diagnosis or a random date (non-ET patients); post-index follow-up was 24 months.

**Results:**

There were 32,984 ET patients (n = 22,641 commercial; n = 10,343 Medicare) and 7,588,080 non-ET patients (n = 7,158,471 commercial; n = 429,609 Medicare). ET patients in both commercial and Medicare populations filled a numerically greater number of unique medications, had a higher numerical prevalence of comorbidities (ie, anxiety, depression, falls), and had numerically greater HCRU and costs than non-ET patients. Most of these numerical trends increased commensurately with increasing number of ET medications.

**Conclusions:**

Compared to non-ET patients, ET patients have higher healthcare costs and utilization, which positively correlated with the number of ET medications. ET patients often have numerically more comorbidities compared to non-ET patients. This analysis demonstrates the medical complexity of ET patients and calls attention to the need for additional therapeutic options.

## Introduction

Essential tremor (ET) is one of the most prevalent neurologic disorders in the United States (US) [1]. ET is characterized by postural and kinetic tremor, usually affecting the upper limbs, but it may also affect the head, lower limbs, vocal cords, trunk, or face [2,3,4,5,6,7]. Although ET is often sporadic, patients with ET who are seen in clinics frequently report a family history of tremor; first-degree relatives of patients with ET are 5 times more likely to develop ET compared to the general population and 10 times more likely to develop ET if tremors manifested at a younger age in the proband with ET [2].

Despite the high incidence [8] and prevalence of ET, first-line treatment options of ET are limited to propranolol and primidone [9,10]. In a previous survey-based study evaluating medication usage conducted among 223 patients with ET treated by movement disorder neurologists, 70.9% of patients reported use of propranolol or primidone; however, 56.3% of the patients receiving propranolol or primidone discontinued treatment. Side effects and lack of efficacy were the most common reasons for discontinuation [11]. Similarly, in a survey conducted by the International Essential Tremor Foundation, the most commonly prescribed medications for ET included beta blockers (42%) and primidone (20%); a total of 35% of patients receiving beta blockers and 23% of patients receiving primidone discontinued use due to side effects. In addition, 34% of patients discontinued use of beta blockers due to lack of efficacy, and 18% discontinued primidone [12]. In a retrospective claims database study of ET patients in the US from 2015 to 2019, propranolol and primidone were the 2 most common ET prescriptions, although discontinuation rates over 2 years were 40% for propranolol and 47% for primidone [13].

Discontinuation of first-line therapy due to lack of efficacy is common among patients with ET, as only 30% to 60% of patients report a reduction in tremor symptoms with pharmacologic therapy [14]. Patients with refractory tremor are often left with invasive options such as deep brain stimulation (DBS) surgery or magnetic resonance-guided focused ultrasound [14].

Current literature regarding treatment efficacy and guideline recommendations reflect an unmet need for pharmacologic and non-pharmacologic ET treatment. Many patients have contraindications or intolerance to current pharmacologic treatment options, are burdened by polypharmacy, or are unwilling or unable to undergo invasive surgical procedures of the brain. To better understand the correlates of ET pharmacotherapy across a large, diverse patient population, a retrospective cohort study was conducted. The objective of the study was to compare the economic burden, including healthcare resource utilization (HCRU) and costs, and comorbidities between subgroups of adult patients with ET taking commonly prescribed ET medications (0, 1, 2, and ≥3) and non-ET patients.

## Methods

### Study design and data source

This study was a retrospective, cohort claims database analysis using the Merative MarketScan^®^ Commercial and Medicare Supplemental Databases to identify the patient population of interest.

The Merative MarketScan Commercial and Medicare Supplemental Databases are part of the Merative MarketScan Research Databases, which consist of deidentified patient-level health data, productivity measures, laboratory results, health risk assessments, hospital discharges, and electronic medical records. There are 3 core claims databases: commercial, Medicare, and multistate Medicaid. Data contributors include employers, managed care organizations, hospitals, providers, Medicare, and Medicaid. Merative MarketScan Research Databases have captured US data on more than 37 billion service records and have more than 120 contributing employers and 40 contributing health plans [15].

This study was not submitted for institutional review board review as it is exempt based on the utilization of secondary, unidentifiable data.

### Study period

The patient identification period was between January 2017 and June 2020 and was the time during which patients with and without ET were identified. For patients with ET, the index date was defined as the first diagnosis date of ET; for those without ET, a random index date was assigned between January 1, 2017 and June 30, 2020.

The post-index follow-up period was 24 months after the index date. As the last qualified index date was January 31, 2020, the full study period was January 1, 2017 through January 31, 2022.

### Patients

Patients included in the study were part of the Merative MarketScan Commercial or Medicare Supplemental Databases during the identification period and required to have 2-years of follow-up claims data. Patients were categorized into commercially insured (aged 22 to <65 years) or Medicare (aged ≥65 years) cohorts. There were 3 main patient groups within the 2 insurance categories: patients with ET who received no pharmacotherapy, patients with ET on drug therapy (ie, 1 ET medication, 2 ET medications, or ≥3 ET medications), and non-ET patients. For the diagnosis of ET, enrollees required ≥1 non-rule-out claim with an International Classification of Diseases, Tenth Revision [ICD-10] diagnosis code of G25.0 during the identification period. Non-rule-out would exclude a claim with a diagnosis of G25.0 that was assigned when a provider was conducting a test to determine whether or not a patient had ET. If the test came back negative, then there would not be subsequent G25.0 codes in that patient’s future, and the enrollee would be classified as non-ET.

Medications that were considered ET-qualified medications in this study included propranolol, primidone, gabapentin, topiramate, and atenolol [9,16,17,18,19,20,21,22]. Patients receiving ET medications that were considered part of the analysis (ie, qualified treatment) had to have been on the medication for ≥31 days or been on overlapping qualified ET medications that equated to ≥31 days. Individuals with less than 31 days of an ET medication during the study were dropped from the analysis. Non-ET patients were classified as individuals who had no claims with a diagnosis of ET at any time during the study period.

### Outcomes

Demographics, including age, sex, and geographic region, were measured on the index date. Data on clinical characteristics (ie, Charlson comorbidity index [23]) and prevalence of claims with diagnoses for anxiety, depression, falls, and substance abuse and treatments (ie, ET-related medications and total number of prescription medications) were assessed in the 2-year period following the index date for each patient. A standard set of ICD-10 codes was used to define the conditions during follow-up or to identify visits with those conditions’ diagnosis codes during the follow-up period consistent with anxiety, depression, falls, or substance abuse disorders.

Additional endpoints assessed in this study included all-cause HCRU, all-cause healthcare costs, and total pharmacotherapy burden. All-cause HCRU was measured during the 24-month post-index period and consisted of inpatient admissions (including length of stay) and emergency department (ED) visits (including number of patients and number of visits per patient). All-cause healthcare costs were measured during the 24-month post-index period and consisted of pharmacy costs and total costs (sum of total medical costs and outpatient pharmacy costs). There is not a standard definition for polypharmacy and/or calculation of pharmacotherapy burden [24]. In this study, total pharmacotherapy burden was determined by evaluating the most commonly filled generic medications in the MarketScan population, and generating indicator flags for the top 50 medications. Total burden then summed up how many of the top 50 medications each ET or non-ET enrollee had during the 2-year follow-up period. We chose this method in order to help identify the total pharmacotherapy burden without including outliers (i.e., without counting rare medications that would inflate the total burden).

### Statistical analysis

Descriptive statistics were used to analyze the results. Means, medians, and standard deviations (SDs) were calculated for each of the endpoints described in the previous section. Non-inflation adjusted twenty-four-month post-index period payment information captured was reported as a mean. When analyzing the presence of events or medications, proportions were calculated for those with an event. The observed differences in values between groups are referred to as being “numerically” lower or higher, as statistical testing was not performed.

## Results

**Figure 1:**
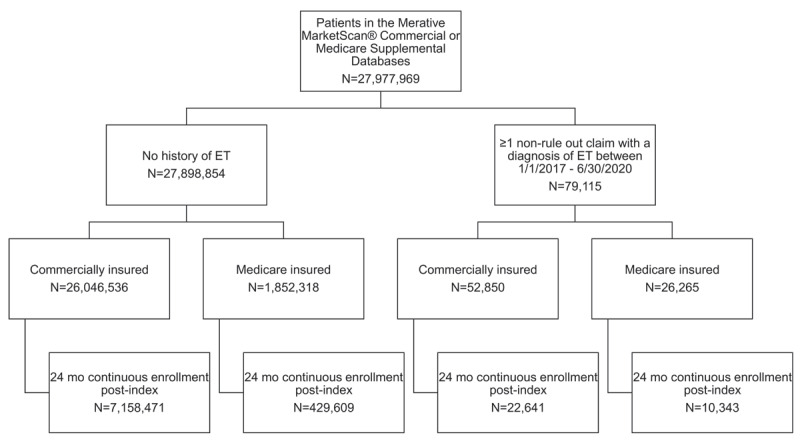
Patient attrition. Key: ET – essential tremor; Mo – months.

A total of 32,984 patients were included in the ET study population (commercial, n = 22,641; Medicare, n = 10,343) and 7,588,080 patients were included in the non-ET study population (commercial, n = 7,158,471; Medicare, n = 429,609) (Figure 1). The majority of patients resided in the South Atlantic, East North Central, and Middle Atlantic geographic regions. Within the entire ET study population, approximately half of patients did not receive ET pharmacotherapy during follow-up. Among commercially insured ET patients, 11,052 (49%) received 0 ET medications, 10,195 (45%) received 1 ET medication, 1,274 (6%) received 2 ET medications, and 120 (<1%) received ≥3 ET medications. Among Medicare ET patients, 6,061 (59%) received 0 ET medications, 3,617 (35%) received 1 ET medication, 597 (6%) received 2 ET medications, and 68 (<1%) received ≥3 ET medications.

### Commercial population (aged 22 to <65 years)

#### Demographics and clinical characteristics

The commercial study population included 22,641 ET patients and 7,158,471 non-ET patients. The ET commercial study population had a mean age of 51.6 years and 51.5% were women (n = 11,648), while the non-ET commercial study population had a mean age of 44.3 years and 53.5% were women (n = 3,829,682) (Table 1).

**Table 1:** Demographics and clinical characteristics.


COMMERCIAL POPULATION						

	ALL ET PATIENTS n = 22,641	0 QUALIFIED ET TREATMENTS n = 11,052	1 QUALIFIED ET TREATMENT n = 10,195	2 QUALIFIED ET TREATMENTS n = 1,274	3+ QUALIFIED ET TREATMENTS n = 120	ALL NON-ET PATIENTS n = 7,158,471

Age, mean (SD; median)	51.6 (9.9; 54.0)	50.9 (10.4; 54.0)	52.1 (9.5; 55.0)	53.7 (8.4; 56.0)	53.6 (7.9; 56.0)	44.3 (11.6; 45.0)

Age category, n (%)						

22–34	1,801 (8.0)	1,059 (9.6)	692 (6.8)	47 (3.7)	3 (2.5)	1,722,197 (24.1)

35–44	2,890 (12.8)	1,518 (13.7)	1,228 (12.1)	130 (10.2)	14 (11.7)	1,717,093 (24.0)

45–54	6,679 (29.5)	3,180 (28.8)	3,101 (30.4)	361 (28.3)	37 (30.8)	1,990,354 (27.8)

55–64	11,271 (49.8)	5,295 (47.9)	5,174 (50.8)	736 (57.8)	66 (55.0)	1,728,827 (24.2)

Sex, n (%)						

Male	10,993 (48.6)	5,673 (51.3)	4,764 (46.7)	518 (40.7)	38 (31.7)	3,328,789 (46.5)

Female	11,648 (51.5)	5,379 (48.7)	5,431 (53.3)	756 (59.3)	82 (68.3)	3,829,682 (53.5)

Geographic region, n (%)						

New England	735 (3.3)	409 (3.7)	287 (2.8)	36 (2.8)	3 (2.5)	268,435 (3.8)

Middle Atlantic	3,062 (13.5)	1,729 (15.6)	1,193 (11.7)	129 (10.1)	11 (9.2)	943,474 (13.2)

East North Central	4,168 (18.4)	2,077 (18.8)	1,834 (18)	236 (18.5)	21 (17.5)	1,199,836 (16.8)

West North Central	1,140 (5)	518 (4.7)	532 (5.2)	76 (6)	14 (11.7)	353,120 (4.9)

South Atlantic	6,663 (29.4)	3,096 (28)	3,149 (30.9)	378 (29.7)	40 (33.3)	2,141,391 (29.9)

East South Central	1,848 (8.2)	790 (7.2)	893 (8.8)	153 (12)	12 (10)	49,7597 (7)

West South Central	2,047 (9)	905 (8.2)	1,016 (10)	121 (9.5)	5 (4.2)	677,263 (9.5)

Mountain	1,405 (6.2)	652 (5.9)	665 (6.5)	79 (6.2)	9 (7.5)	452,371 (6.3)

Pacific	1,535 (6.8)	866 (7.8)	602 (5.9)	62 (4.9)	5 (4.2)	608,616 (8.5)

Unknown/Missing	38 (0.2)	10 (0.1)	24 (0.2)	4 (0.3)	0 (0)	16,368 (0.2)

CCI^a^, mean (SD; median)	1.0 (1.8; 0.0)	0.9 (1.6; 0.0)	1.2 (1.9; 0.0)	1.6 (2.2; 1.0)	1.9 (2.2; 1.0)	0.5 (1.1; 0.0)

ET-related medications with ≥31 days’ supply, n (%)	11,589 (51.2)	0 (0.0)	10,195 (100.0)	1,274 (100.0)	120 (100.0)	409,170 (5.7)

Atenolol	620 (5.3)	NA	473 (4.6)	117 (9.2)	30 (25.0)	83,246 (20.3)

Gabapentin	2,127 (18.4)	NA	1,445 (14.2)	591 (46.4)	91 (75.8)	183,902 (44.9)

Primidone	2,636 (22.7)	NA	1,862 (18.3)	683 (53.6)	91 (75.8)	845 (0.2)

Propranolol	6,421 (55.4)	NA	5,535 (54.3)	808 (63.4)	78 (65.0)	61,828 (15.1)

Topiramate	1,306 (11.3)	NA	880 (8.6)	349 (27.4)	77 (64.2)	89,573 (21.9)

Days on LOT, mean (SD; median)	432.8 (229.0; 448.1)	NA	445.5 (454.2; 241.0)	350.1 (396.9; 180.0)	234.6 (262.1; 136.0)	369.1 (399.3; 180.0)

Number of unique top 50 NDCs, mean (SD; median)	6.6 (4.5; 6.0)	5.2 (4.0; 4.0)	7.7 (4.4; 7.0)	10.2 (5.0; 10.0)	12.6 (5.3; 12.0)	3.6 (3.4; 3.0)

**MEDICARE POPULATION**						

	**ALL ET PATIENTS n = 10,343**	**0 QUALIFIED ET TREATMENTS n = 6,061**	**1 QUALIFIED ET TREATMENT n = 3,617**	**2 QUALIFIED ET TREATMENTS n = 597**	**3+ QUALIFIED ET TREATMENTS n = 68**	**ALL NON-ET PATIENTS n = 429,609**

Age, mean (SD; median)	75.7 (7.0; 75.0)	75.5 (7.2; 74.0)	75.5 (7.1; 74.0)	75.1 (6.6; 75.0)	75.8 (7.0; 75.0)	74.0 (7.1; 72.0)

Age category, n (%)						

65–74	5,091 (49.2)	2,940 (48.5)	1,820 (50.3)	299 (50.1)	32 (47.1)	256,793 (59.8)

75+	5,252 (50.8)	3,121 (51.5)	1,797 (49.7)	298 (49.9)	36 (52.9)	172,816 (40.2)

Sex, n (%)						

Male	4,837 (46.8)	2,846 (47.0)	1,693 (46.8)	269 (45.1)	29 (42.7)	184,833 (43.0)

Female	5,506 (53.2)	3,215 (53.0)	1,924 (53.2)	328 (54.9)	39 (57.4)	244,776 (57.0)

Geographic region, n (%)						

New England	173 (1.7)	82 (1.4)	76 (2.1)	13 (2.2)	2 (2.9)	7,250 (1.7)

Middle Atlantic	2,814 (27.2)	1,442 (23.8)	1,182 (32.7)	177 (29.7)	13 (19.1)	117,788 (27.4)

East North Central	3,889 (37.6)	2,870 (47.4)	862 (23.8)	141 (23.6)	16 (23.5)	176,627 (41.1)

West North Central	94 (0.9)	45 (0.7)	38 (1.1)	8 (1.3)	3 (4.4)	3,790 (0.9)

South Atlantic	1941 (18.8)	944 (15.6)	828 (22.9)	148 (24.8)	21 (30.9)	70,261 (16.4)

East South Central	168 (1.6)	72 (1.2)	79 (2.2)	16 (2.7)	1 (1.5)	6,226 (1.5)

West South Central	389 (3.8)	164 (2.7)	186 (5.1)	33 (5.5)	6 (8.8)	12,997 (3)

Mountain	588 (5.7)	307 (5.1)	240 (6.6)	37 (6.2)	4 (5.9)	23,156 (5.4)

Pacific	277 (2.7)	132 (2.2)	119 (3.3)	24 (4)	2 (2.9)	11,087 (2.6)

Unknown/Missing	10 (0.1)	3 (0.1)	7 (0.2)	0 (0)	0 (0)	427 (0.1)

CCI^a^, mean (SD; median)	2.7 (2.6; 2.0)	2.6 (2.6; 2.0)	2.7 (2.6; 2.0)	3.1 (2.8; 3.0)	3.0 (2.4; 3.0)	1.9 (2.4; 1.0)

ET-related medications with ≥31 days’ supply, n (%)	4,282 (41.4)	0 (0.0)	3,617 (100.0)	597 (100.0)	68 (100.0)	41,562 (9.7)

Atenolol	380 (8.9)	NA	283 (7.8)	77 (12.9)	20 (29.4)	15,311 (36.8)

Gabapentin	1,044 (24.4)	NA	719 (19.9)	274 (45.9)	51 (75.0)	22,927 (55.2)

Primidone	1,531 (35.8)	NA	1,072 (29.6)	401 (67.2)	58 (85.3)	264 (0.6)

Propranolol	1,763 (41.2)	NA	1,367 (37.8)	352 (59.0)	44 (64.7)	2,589 (6.2)

Topiramate	299 (7.0)	NA	176 (4.9)	90 (15.1)	33 (48.5)	1,509 (3.6)

Days on LOT, mean (SD; median)	488.8 (301.5; 462.9)	NA	479.6 (285.0; 470.5)	423.0 (269.0; 415.0)	249.4 (126.0; 283.3)	484.6 (437.6; 301.0)

Number of unique top 50 NDCs, mean (SD; median)	7.6 (4.1; 7.0)	6.9 (4.0; 6.0)	8.6 (4.1; 8.0)	9.8 (4.1; 10.0)	10.4 (3.6; 10.0)	5.6 (3.7; 5.0)


^a^ The CCI is a tool that assigns morbidity scores that reflect mortality risk to patients based upon medical conditions and was determined using a weighted value based on 1 or more diagnosis or procedure codes during follow-up for the individual conditions listed in the CCI [23]. Key: CCI – Charlson Comorbidity Index; ET – essential tremor; LOT – line of therapy; NA ‒ not available; NDC ‒ National Drug Code; SD ‒ standard deviation.

**Figure 2:**
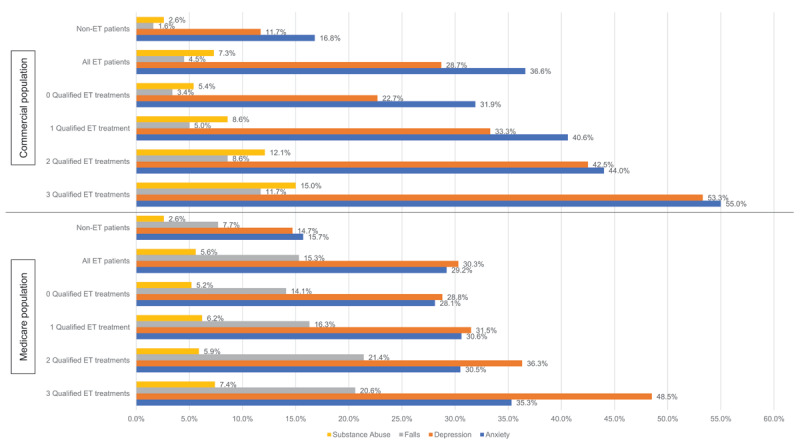
Comorbid diagnoses. Key: ET – essential tremor.

In the overall ET population, the mean (SD; median) Charlson Comorbidity Index (CCI) score was 1 (1.8; 0.0) and ranged from 0.9 (1.6; 0.0) in patients not receiving medications up to 1.9 (2.2; 1.0) in patients receiving ≥3 ET medications; the most prevalent clinical condition reported was anxiety (n = 8,293 [36.6%]), followed by depression (n = 6,506 [28.7%]), falls (n = 1,014 [4.5%]), and substance abuse (n = 1,646 [7.3%]). Numerically, ET patients receiving ≥3 ET medications had higher rates of anxiety, depression, falls, and substance abuse (Table 1; Figure 2). The mean (SD; median) CCI score in non-ET patients was 0.5 (1.1; 0.0), which is numerically lower than those with ET. Similar to patients with ET, the most prevalent clinical conditions reported in non-ET patients were anxiety (n = 1,205,645 [16.8%]), depression (n = 839,252 [11.7%]), falls (114,661 [1.6%]), and substance abuse (n = 184,511 [2.6%]) (Table 1; Figure 2), although all proportions of these conditions were numerically lower in non-ET patients than in ET patients.

#### All-cause HCRU and healthcare costs

**Figure 3:**
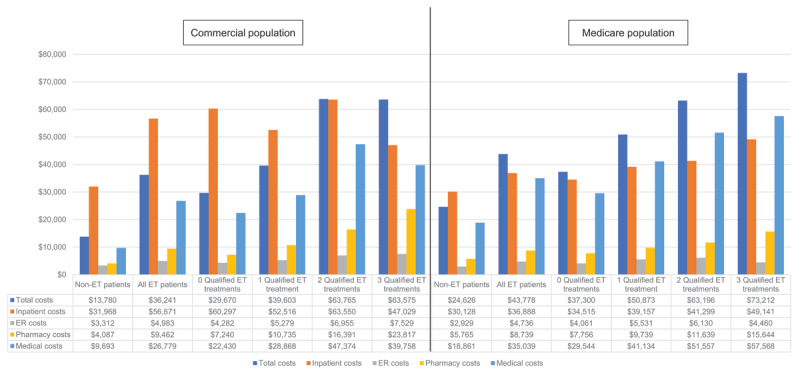
Mean all-cause costs. Key: ER – emergency room; ET – essential tremor.

During the 2-year post-index period, 15.3% (n = 3,465/22,641) of ET patients had an inpatient admission, with a mean (SD; median) length of stay of 9.6 (21.6; 4.0) days and a mean (SD; median) cost of $56,671 ($118,584; $28,107). Inpatient admission rates were numerically highest in patients receiving ≥3 ET medications. However, the numerically highest costs and longest lengths of stay were seen in those patients receiving 2 ET medications. There were 582,100 (n = 7,158,471; 8.1%) non-ET patients who had an inpatient admission during the 2-year follow-up period. The mean (SD; median) number of inpatient admissions per non-ET patient was 1.3 (0.9; 1.0) and the mean (SD; median) inpatient length of stay (in days) was 4.9 (9.9; 2.0). The mean (SD; median) inpatient cost per non-ET patient was $31,968 ($59,221; $16,952) (Table 2; Figure 3).

**Table 2:** All-cause healthcare resource utilization and costs.


COMMERCIAL POPULATION						

	All ET PATIENTS n = 22,641	0 QUALIFIED ET TREATMENTS n = 11,052	1 QUALIFIED ET TREATMENT n = 10,195	2 QUALIFIED ET TREATMENTS n = 1,274	3+ QUALIFIED ET TREATMENTS n = 120	ALL NON-ET PATIENTS n = 7,158,471

Inpatient admission, n, (%)	3,465 (15.3)	1,390 (12.6)	1,701 (16.7)	341 (26.8)	33 (27.5)	582,100 (8.1)

No., mean (SD)	1.7 (1.7)	1.7 (1.8)	1.7 (1.4)	2.1 (2.9)	1.6 (1.1)	1.3 (0.9)

LOS, days, mean (SD)	9.6 (21.6)	9.1 (23.2)	9.5 (18.8)	12.6 (28.1)	7.9 (9.7)	4.9 (9.9)

Cost, mean (SD; median)	$56,671 (118,584; 28,107)	$60,297 (146,119; 27,041)	$52,516 (85,522; 28,672)	$63,550 (138,039; 29,833)	$47.029 (48,560; 32,176)	$31,968 (59,221; 16,952)

ED visit, n (%)	7,615 (33.6)	3,343 (30.2)	3,650 (35.8)	575 (45.1)	47 (39.2)	1,598,837 (22.3)

No., mean (SD)	2.4 (3.5)	2.1 (2.5)	2.4 (3.1)	3.4 (7.9)	3.6 (4.6)	1.8 (2.0)

Cost, mean (SD; median)	$4,983 (9,746; 2,375)	$4,282 (7,294; 2,152)	$5,279 (9,144; 2,533)	$6,955 (20,079; 3,282)	$7,529 (8,930; 4,826)	$3,312 (6,526; 1,733)

Pharmacy costs, mean (SD; median)	$9,462 (30,321; 1,591)	$7,240 (27,422; 872)	$10,735 (32,165; 2,129)	$16,391 (34,561; 5,413)	$23,817 (41,592; 8,441)	$4,087 (23,854; 274)

Total costs, mean (SD; median)	$36,241 (86,884; 12,326)	$29,670 (84,700; 9,439)	$39,603 (80,849; 14,773)	$63,765 (133,880; 28,826)	$63,575 (66,440; 29,032)	$13,780 (44,521; 3,383)

**MEDICARE POPULATION**						

	**ALL ET PATIENTS n = 10,343**	**0 QUALIFIED ET TREATMENTS n = 6,061**	**1 QUALIFIED ET TREATMENT n = 3,617**	**2 QUALIFIED ET TREATMENTS n = 597**	**3+ QUALIFIED ET TREATMENTS n = 68**	**All NON-ET PATIENTS n = 429,609**

Inpatient admissions (n, %)	3,312 (32.0)	1,797 (29.6)	1,236 (34.2)	250 (41.9)	29 (42.6)	80,475 (18.7)

No., mean (SD)	1.6 (1.2)	1.6 (1.4)	1.6 (1.0)	1.7 (1.1)	1.5 (0.8)	1.4 (0.9)

LOS, days, mean (SD)	7.8 (13.7)	7.9 (15.2)	7.6 (11.1)	8.5 (14.9)	6.5 (4.1)	6.4 (10.5)

Cost, mean (SD; median)	$36,888 (68,056; 16,359)	$34,515 (63,396; 16,087)	$39,157 (73,464; 16,181)	$41,299 (69,164; 20,100)	$49,141 (91,610; 18,015)	$30,128 (61,242; 13,039)

ED visit (n, %)	4,858 (47.0)	2,732 (45.1)	1,771 (49.0)	319 (53.4)	36 (52.9)	136,912 (31.9)

No. (mean, SD)	2.6 (2.6)	2.7 (2.8)	2.6 (2.4)	2.6 (2.5)	2.3 (1.6)	2.1 (2.1)

Cost, mean (SD; median)	$4,736 (10,896; 1,403)	$4,061 (9,241; 1,294)	$5,531 (12,524; 1,520)	$6,130 (13,921; 1,785)	$4,460 (5,635; 2,441)	$2,929 (7,032; 850)

Pharmacy costs, mean (SD; median)	$8,739 (25,180; 2,714)	$7,756 (24,413; 1,944)	$9,739 (26,293; 3,731)	$11,639 (25,331; 5,675)	$15,644 (24,562; 6,300)	$5,765 (20,612; 1,092)

Total costs, mean (SD; median)	$43,778 (81,894; 19,141)	$37,300 (68,867; 16,218)	$ 50,873 (95,858; 22,295)	$ 50,873 (95,858; 22,295)	$73,212 (97,801; 34,147)	$24,626 (64,431; 8,380)


Key: ED ‒ emergency department; ET – essential tremor; NA ‒ not available; No. – number; SD ‒ standard deviation.

ED visits: The percentage of ET patients with an ED visit was 33.6% (n = 7,615/22,641), with a mean (SD; median) cost of $4,983 ($9,746; $2,375). The percentage of patients with an ED visit and costs increased with increasing number of ET treatments, with those patients taking 2 ET medications experiencing the numerically highest rate of ED visits at 45.1% (n = 575/1,274).

Total costs (total medical and pharmacy costs): The mean (SD; median) pharmacy cost per ET patient was $9,462 ($30,321; $1,591). Patients receiving ≥3 ET medications had the numerically highest pharmacy cost per patient. The mean (SD; median) total cost per patient was $36,241 ($86,884; $12,326). Mean total costs (SD; median) were numerically highest in patients receiving 2 ET medications ($63,765 [$133,880; $28,826]). In non-ET patients, the mean (SD; median) pharmacy cost per patient was $4,087 ($23,854; $274) and the mean (SD; median) total cost per patient was $13,780 ($44,521; $3,383).

#### ET-related treatment patterns

**Figure 4:**
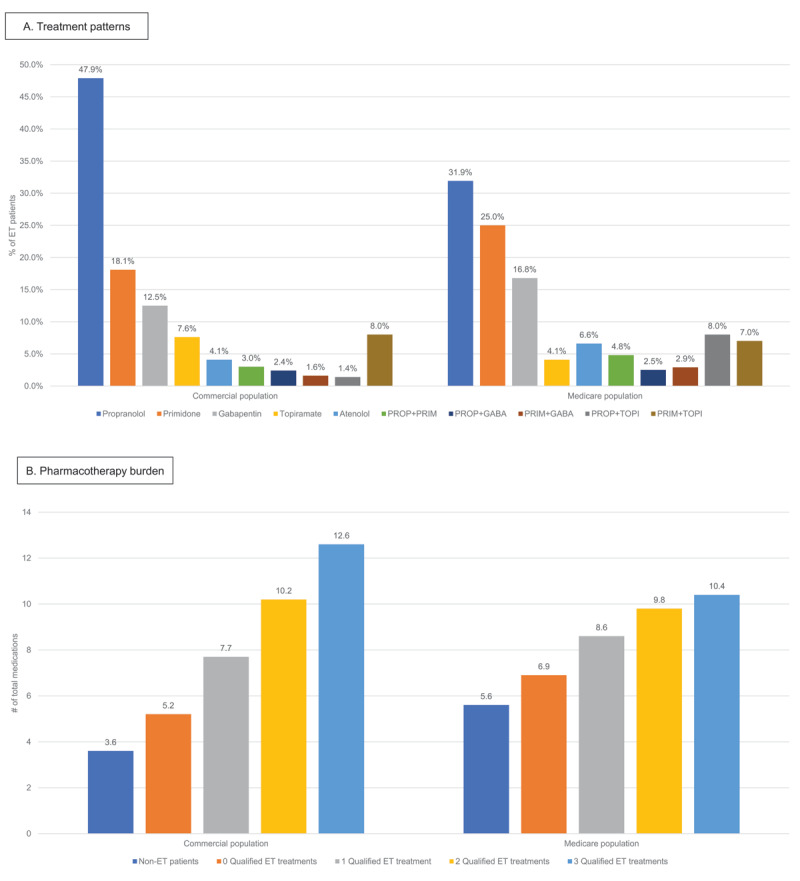
Treatment patterns **(A)** and pharmacotherapy burden **(B)**. Key: ET – essential tremor; GABA – gabapentin; PRIM – primidone; PROP – propranolol; TOPI – topiramate.

The 3 most commonly filled ET treatments over the post-index period were propranolol (n = 5,535/11,589 [47.8%]), primidone (n = 1,862/11,589 [16.1%]), and gabapentin (n = 1,445/11.589 [12.5%]). The mean days of exposure were 489.9, 435.1, and 283.3 days, respectively. The 5 most common ET treatment combinations were propranolol and primidone (n = 346; 3.0%), propranolol and gabapentin (n = 282; 2.4%), primidone and gabapentin (n = 190; 1.6%), propranolol and topiramate (n = 159; 1.4%), and primidone and topiramate (n = 94; 0.8%) (Figure 4A).

#### Total pharmacotherapy burden

In ET patients, the mean number of uniquely named generic prescriptions filled at least once during follow-up per patient were 6.6 and increased with increasing number of ET medications (5.2, 7.7, 10.2, and 12.6 in patients take 0, 1, 2, or 3+ ET medications, respectively); this was numerically higher than non-ET patients who were only receiving a mean of 3.6 unique prescriptions per patient (Figure 4B).

### Medicare population (aged ≥65 years)

#### Demographics and clinical characteristics

The Medicare study population included 10,343 ET patients and 429,609 non-ET patients (Table 1).The ET Medicare study population had a mean age of 75.7 years and 53.2% were women (n = 5,506), while the non-ET Medicare study population had a mean age of 74 years and 57.0% were women (n = 244,776).

The mean (SD; median) CCI score for the ET population was 2.7 (2.6; 2.0) and increased up to 3.1 (2.8; 3.0) in patients receiving 2 ET medications. Among reported conditions, the most common claims were for depression (n = 3,134 [30.3%]), anxiety (n = 3,017 [29.2%]), falls (n = 1,587 [15.3%]), and substance abuse (n = 578 [5.6%]). Numerically, ET patients receiving ≥3 ET medications had the highest rates of depression, anxiety, falls, and substance abuse (Figure 2). The mean (SD; median) CCI score for the non-ET population was 1.9 (2.4; 1.0). Similar to ET patients, but numerically lower in post-index prevalence, the most common clinical condition reported was anxiety (n = 67,382 [15.7%]), followed by depression (n = 63,256 [14.7%]), falls (n = 33,228 [7.7%]), and substance abuse (11,128 [2.6%]).

#### All-cause HCRU and cost

Inpatient Admissions: During the 2-year post-index period, 32.0% (n = 3,312/10,343) of ET patients had an inpatient admission, with a mean (SD; median) cost per patient of $36,888 ($68,056; $16,539). The mean (SD; median) inpatient length of stay per patient was 7.8 days (13.7; 4.0). Admission rates were numerically highest in patients receiving ≥3 ET medications, while length of stay was numerically longest in patients receiving 2 ET medications. During the post-index period, 18.7% (n = 80,475/429,609) of non-ET patients had an inpatient admission, with a mean (SD; median) number of inpatient admissions per patient of 1.4 (0.9; 1.0) and a mean (SD; median) inpatient length of stay (in days) of 6.4 (10.5; 4.0). The mean (SD; median) inpatient cost per non-ET patient was $30,128 ($61,242; $13,039) (Table 2).

ED visits: The percentage of patients with an ED visit was 47.0% (n = 4,858/10,343); the mean (SD; median) ED visit cost per patient was $4,736 ($10,896; $1,403). Patients receiving 2 ET medications had the numerically highest rate, number, and cost of ED visits.

Total costs (total medical and pharmacy costs): The mean (SD; median) pharmacy cost was $8,739 ($25,180; $2,714) and the mean (SD; median) total cost per patient was $43,778 ($81,894; $19,141). Mean total costs were numerically highest among those ET patients receiving ≥3 ET medications ($73,212 [$97,801; $34,147]). In non-ET patients, the mean (SD; median) pharmacy cost per patient was $5,765 ($20,612; $1,092) and the mean (SD; median) total cost was $24,626 ($64,431; $8,380).

#### ET-related treatment patterns

The 3 most common ET treatments were propranolol (n = 1,367/4,282 [31.9%]), primidone (n = 1,072/4,282 [25.0%]), and gabapentin (n = 719/4,282 [16.8%]). The mean days of exposure were 553.3, 514.4, and 342.8 days, respectively. The 5 most common ET treatment combinations were propranolol and primidone (n = 205; 4.8%), primidone and gabapentin (n = 125; 2.9%), primidone and atenolol (n = 39; 0.9%), propranolol and topiramate (n = 36; 0.8%), and primidone and topiramate (n = 32; 0.7%) (Figure 4A).

#### Total pharmacotherapy burden

In ET patients, the average number of uniquely named generic prescriptions filled at least once during follow-up per patient was 7.6 and numerically increased with increasing number of ET medications (6.8, 8.5, 9.8, and 10.5 in patients taking 0, 1, 2, or 3+ ET medications, respectively); this was numerically higher than non-ET patients who were only receiving an average of 5.5 unique prescriptions (Figure 4B).

## Discussion

In this study, ET patients compared to non-ET patients in both commercial and Medicare populations were taking a numerically greater number of total medications, had a numerically higher prevalence of ET-associated clinical conditions (ie, anxiety, depression, falls, and substance abuse), and numerically greater HCRU and costs. Most of these trends increased with receipt of increasing number of ET medications, which could be due to multiple reasons including the medical complexity of ET patients.

In our study, approximately 13% of patients on ET treatment required 2 or more ET medications, highlighting a population of patients in whom 1 medication may not be optimal. Patients who took additional ET medications often had numerically higher rates of comorbid psychiatric conditions such as depression, anxiety, and substance abuse, which could reflect the burden of ET, as it is a chronic, progressive disease. This trend was noted in both the Medicare and commercial populations. A retrospective observational study noted similar results when comparing rates of depression and anxiety between patients with ET (n = 5,286) and patients without ET (n = 5,244). Patients with ET had higher rates of depression (20.2% vs 1.3%; *P* < 0.001) and anxiety (22.2% vs 1.34%; *P* < 0.001) [25]. Other studies have similarly noted greater medical and psychiatric comorbidity in ET patients than controls [26].

Similarly, in an alternative analysis of the Merative MarketScan Commercial and Medicare Supplemental Databases (1/1/2017–12/31/2018), patients with ET were diagnosed with depression or anxiety within the first 24 months after the first ET claim more frequently than patients without ET in both commercially and Medicare-covered individuals [27]. The proportion of patients with depression ranged from 27.6% to 31.7% of ET patients vs 11.0% to 14.2% of non-ET patients, and anxiety proportions ranged from 34.1% to 39.3% of ET patients vs 14.4% to 14.8% of non-ET patients [27]. In a retrospective claims database study of ET patients in the US from 2015 to 2019, rates of depression and anxiety increased in the 48 months prior to ET diagnosis and peaked just prior to ET diagnosis, from 31% to 42% and 10% to 20%, respectively. Patients with a confirmed diagnosis of ET in 2019 had depression and anxiety rates of 44% and 29%, respectively [13]. Our rates may be higher than those reported by the other studies discussed [13,25,27] due to measurement in the first year of ET diagnosis. Taken together, these results further highlight the frequency and complexity of psychiatric comorbidities in patients with ET.

As seen in this study, patients with ET who were taking 2 ET medications took an average of 10 unique medications for multiple conditions, including ET (10.2 for commercial, 9.8 for Medicare), over the 2-year follow-up period, indicating a substantial pharmacotherapy and comorbidity burden and putting patients at potential risk of drug-drug interactions with clinical and economic consequences. This trend has been reported in other studies. In a retrospective cohort study of US patients with ET from 2015 to 2019 (n = 128,263), 96% of patients included in the study had at least 1 comorbidity and 76% of ET-treated patients were receiving concomitant treatment for either cardiometabolic, psychiatric, or movement-related comorbidities [13].

High rates of comorbidities were also reported in a retrospective observational study of US patients. Patients with ET had a higher number of comorbidities than non-ET patients, and many ET patients had multiple comorbidities, with 79.7% having at least 3 comorbidities. Additionally, patients aged 65 years or older had a significantly higher comorbidity burden than those 45 to 64 years old or <45 years (5.6 vs 4.6 vs 2.4; *P* < 0.0001) [25]. Similarly, in this study, the prevalence of comorbidities was observed to be numerically higher in ET patients compared to non-ET patients, as evidenced by higher CCI scores in the ET population, particularly for older patients in the Medicare cohort.

Polypharmacy has a significant impact on patients and our healthcare system. Prior research has shown that patients taking 5 to 9 medications have a 50% chance of an adverse drug reaction and that polypharmacy may contribute to almost 30% of all hospital admissions in the US [28,29]. In a single-center study of 6,545 patients aged 60 years or older, 1.3% of medication lists were found to have a drug-drug interaction. Of those, the average number of medications per patient was 8.6, compared to only 3.1 in patients without a drug-drug interaction. Severity of interactions ranged from contraindicated (2.1%) to generally avoid (27.4%) or monitor closely (70.5%). When extrapolated to the entire Medicare population, authors estimated over 1.7 million patients may have a drug-drug interaction on their medication list [30]. Of note, these results are particularly relevant to our study population as the majority of drug-drug interactions were with psychotropic agents, and the most prevalent clinical conditions in our population were anxiety and depression, for which psychotropic medications are frequently prescribed.

In the previously described retrospective study of US patients, all-cause HCRU, including inpatient admissions (21.02% vs 16.5%; *P* < 0.001), ED visits (30.2% vs 25.0%; *P* < 0.001), length of stay (3.3 days vs 2.7 days; *P* < 0.001), and all-cause costs ($17,560 vs $13,237; *P* < 0.001), was significantly higher in patients with ET compared to those without ET [25]. Similar to our study, higher all-cause HCRU (22.4% vs 21.0% inpatient admissions, 31.8% vs 30.2% ED visits, and mean length of stay of 3.6 days vs 3.3) and higher all-cause costs ($18,647 vs $17,560) were seen in ET patients receiving pharmacotherapy than the overall ET patient population. Even in ET patients who remained untreated, the pharmacotherapy, HCRU, and cost burden was higher when compared to non-ET patients.

Over half of the ET patients in this study (52%) were not treated for ET, pointing to a population with high unmet needs. This population should be investigated further to uncover reasons for lack of treatment or treatment discontinuation so they may be addressed through new or more tolerable treatment interventions to improve disease control and reduce clinical and economic burden.

### Limitations

There are several limitations associated with this study. Retrospective studies inherently have limitations such as lack of randomization or blinding, which lend themselves to increased impact of confounding factors and potential biases on results. For example, we utilized disease codes to identify patients with ET, but we were agnostic to the underlying condition an individual prescription was written for and were unable to verify the prescription was intended for ET treatment. The focus on high impact events (i.e., inpatient admission or ER visits) leaves the room for additional research related more general office-based medical costs, reflecting the non-emergent burden of ET. Statistical models were not completed to formally test whether the numerical differences observed between populations was above and beyond that expected to due chance. The generalizability of the results is also limited as the patient population only consisted of those who were commercially or Medicare-insured. Therefore, results cannot be applied to those who are uninsured. We did not use implantation of DBS as a selection criterion, so patients who have received DBS may have been included. We defined total pharmacotherapy burden based on the use of the top 50 medications list for US patients rather than the total number of medications. A less restrictive list may have yielded different results. An analysis of patients who underwent invasive treatments would have been of additional interest, as these patients may have higher associated healthcare use. Finally, patients with greater health seeking behavior are expected to have higher health care utilization, although we did not assess this quality in these patients. Despite the limitations discussed in this section, this is the first report to our knowledge to assess the total pharmacotherapy burden of patients with ET in the US; therefore, the results from this study are an important addition to the body of ET literature.

## Conclusion

Compared to non-ET patients, ET patients have higher healthcare costs and utilization, which is positively correlated with the number of ET medications. ET patients also often have numerically more comorbidities (ie, anxiety, depression, falls, and substance abuse), which leads to greater need for medical care. Older patients have an added layer of complexity with a higher prevalence of comorbidities, in addition to greater pharmacotherapy burden owing to treatment of those comorbidities. While ET has been traditionally regarded as benign, this analysis demonstrates the medical complexity of ET patients. It also calls attention to the need for additional therapeutic options.

## Additional Files

The additional files for this article can be found as follows:

10.5334/tohm.973.s1 Supplementary Table 1. Distribution of most frequent top 50 prescriptions.

10.5334/tohm.973.s2 Supplementary Table 2. ET-related healthcare resource utilization and costs.
