# Supplementary material for: Clinical and Economic Correlates of Pharmacotherapy in Patients with Essential Tremor
Source: Tremor Other Hyperkinet Mov (N Y). 2024 Dec 17;14:60. doi: 10.5334/tohm.973 (PMC11661014; doi:10.5334/tohm.973)
Supplement: Supplementary Table 1. — Distribution of most frequent top 50 prescriptions. [file tohm-14-1-973-s1.pdf]

S Table 1. Distribution of most frequent top 50 prescriptions

Commercial population						
**Prescription (n, %)**	**All ET patients N=22,641**	**0 qualified ET treatments n=11,052**	**1 qualified ET treatment n=10,195**	**2 qualified ET treatments n=1,274**	**3+ qualified ET treatments n=120**	**All non-ET patients n=7,158,471**
Propranolol hydrochloride	8,021 (35.4)	834 (7.5)	6,234 (61.1)	870 (68.3)	83 (69.2)	96,421 (1.3)
Prednisone	5,843 (25.8)	2,460 (22.3)	2,899 (28.4)	431 (33.8)	53 (44.2)	1,206,960 (16.9)
Azithromycin	5,515 (24.4)	2,487 (22.5)	2,595 (25.5)	385 (30.2)	48 (40.0)	1,300,377 (18.2)
Acetaminophen/hydrocodone bitartrate	5,236 (23.1)	2,063 (18.7)	2,674 (26.2)	444 (34.9)	55 (45.8)	986,536 (13.8)
Influenza virus vaccine (subvirion)	4,623 (20.4)	2,108 (19.1)	2,206 (21.6)	282 (22.1)	27 (22.5)	1,133,808 (15.8)
Albuterol sulfate	4,606 (20.3)	1,963 (17.8)	2,223 (21.8)	373 (29.3)	47 (39.2)	862,736 (12.1)
Amoxicillin	4,580 (20.2)	2,036 (18.4)	2,209 (21.7)	303 (23.8)	32 (26.7)	1,210,034 (16.9)
Amoxicillin/clavulanate potassium	4,539 (20.0)	1,967 (17.8)	2,227 (21.8)	305 (23.9)	40 (33.3)	1,058,596 (14.8)
Methylprednisolone	4,471 (19.7)	1,894 (17.1)	2,231 (21.9)	309 (24.3)	37 (30.8)	874,730 (12.2)
Gabapentin	4,113 (18.2)	687 (6.2)	2,633 (25.8)	699 (54.9)	94 (78.3)	371,688 (5.2)
Atorvastatin calcium	4,110 (18.2)	1,667 (15.1)	2,091 (20.5)	320 (25.1)	32 (26.7)	692,169 (9.7)
Primidone	3,884 (17.2)	378 (3.4)	2,676 (26.2)	737 (57.8)	93 (77.5)	1,220 (0.02)
Fluticasone propionate	3,543 (15.6)	1,554 (14.1)	1,711 (16.8)	255 (20.0)	23 (19.2)	736,566 (10.3)
Cyclobenzaprine hydrochloride	3,531 (15.6)	1,377 (12.5)	1,820 (17.9)	299 (23.5)	35 (29.2)	638,723 (8.9)
Benzonatate	3,235 (14.3)	1,365 (12.4)	1,589 (15.6)	255 (20.0)	26 (21.7)	667,932 (9.3)
Cephalexin	3,191 (14.1)	1,329 (12.0)	1,574 (15.4)	260 (20.4)	28 (23.3)	621,739 (8.7)
Levothyroxine sodium	3,130 (13.8)	1,290 (11.7)	1,551 (15.2)	262 (20.6)	27 (22.5)	567,173 (7.9)
Omeprazole	3,117 (13.8)	1,269 (11.5)	1,577 (15.5)	240 (18.8)	31 (25.8)	493,602 (6.9)
Doxycycline hyclate	3,019 (13.3)	1,272 (11.5)	1,470 (14.4)	252 (19.8)	25 (20.8)	589,746 (8.2)
Meloxicam	2,876 (12.7)	1,183 (10.7)	1,438 (14.1)	234 (18.4)	21 (17.5)	508,465 (7.1)
Ibuprofen	2,869 (12.7)	1,319 (11.9)	1,341 (13.2)	192 (15.1)	17 (14.2)	975,753 (13.6)
Bupropion hydrochloride	2,767 (12.2)	1,002 (9.1)	1,522 (14.9)	220 (17.3)	23 (19.2)	327,605 (4.6)
Lisinopril	2,747 (12.1)	1,197 (10.8)	1,339 (13.1)	194 (15.2)	17 (14.2)	549,224 (7.7)
Tramadol hydrochloride	2,712 (12.0)	989 (8.9)	1,439 (14.1)	256 (20.1)	28 (23.3)	396,188 (5.5)
Ciprofloxacin hydrochloride	2,667 (11.8)	1,089 (9.9)	1,327 (13.0)	225 (17.7)	26 (21.7)	484,312 (6.8)
Sulfamethoxazole/trimethoprim	2,601 (11.5)	1,043 (9.4)	1,331 (13.1)	202 (15.9)	25 (20.8)	516,051 (7.2)
Alprazolam	2,541 (11.2)	1,037 (9.4)	1,278 (12.5)	206 (16.2)	20 (16.7)	335,648 (4.7)
Acetaminophen/oxycodone	2,486 (11.0)	951 (8.6)	1,262 (12.4)	243 (19.1)	30 (25.0)	429,837 (6.0)

hydrochloride						
Metformin hydrochloride	2,380 (10.5)	914 (8.3)	1,237 (12.1)	209 (16.4)	20 (16.7)	504,650 (7.0)
Fluconazole	2,265 (10.0)	886 (8.0)	1,140 (11.2)	212 (16.6)	27 (22.5)	558,172 (7.8)
Amlodipine besylate	2,246 (9.9)	971 (8.8)	1,095 (10.7)	162 (12.7)	18 (15.0)	452,885 (6.3)
Diclofenac sodium	2,175 (9.6)	833 (7.5)	1,113 (10.9)	208 (16.3)	21 (17.5)	366,375 (5.1)
Pantoprazole sodium	2,172 (9.6)	853 (7.7)	1,104 (10.8)	187 (14.7)	28 (23.3)	312,558 (4.4)
Clonazepam	2,146 (9.5)	774 (7.0)	1,161 (11.4)	187 (14.7)	24 (20.0)	141,911 (2.0)
Triamcinolone acetonide	2,144 (9.5)	973 (8.8)	1,001 (9.8)	153 (12.0)	17 (14.2)	451,236 (6.3)
Montelukast sodium	2,143 (9.5)	888 (8.0)	1,069 (10.5)	161 (12.6)	25 (20.8)	362,158 (5.1)
Ondansetron	2,113 (9.3)	848 (7.7)	1,060 (10.4)	187 (14.7)	18 (15.0)	390,521 (5.5)
Trazodone hydrochloride	2,108 (9.3)	738 (6.7)	1,137 (11.20	212 (16.6)	21 (17.5)	224,861 (3.1)
Escitalopram oxalate	2,056 (9.1)	815 (7.4)	1,085 (10.6)	140 (11.0)	16 (13.3)	333,073 (4.7)
Sertraline hydrochloride	2,023 (8.9)	800 (7.2)	1,050 (10.3)	154 (12.1)	19 (15.8)	323,740 (4.5)
Topiramate	2,003 (8.8)	148 (1.3)	1,367 (13.4)	412 (32.3)	76 (63.3)	117,721 (1.6)
Duloxetine hydrochloride	1,892 (8.4)	600 (5.4)	1,063 (10.4)	205 (16.1)	24 (20.0)	154,042 (2.2)
Metoprolol succinate	1,851 (8.2)	1,052 (9.5)	709 (7.0)	82 (6.4)	8 (6.7)	252,096 (3.5)
Ondansetron hydrochloride	1,843 (8.1)	658 (6.0)	991 (9.7)	177 (13.9)	17 (14.2)	301,889 (4.2)
Losartan potassium	1,816 (8.0)	807 (7.3)	863 (8.5)	126 (9.9)	20 (16.7)	349,504 (4.9)
Rosuvastatin calcium	1,773 (7.8)	762 (6.9)	882 (8.7)	114 (8.9)	15 (12.5)	285,044 (4.0)
Metronidazole	1,705 (7.5)	720 (6.5)	865 (8.5)	111 (8.7)	9 (7.5)	424,702 (5.9)
Nitrofurantoin monohydrate/ nitrofurantoin, macro	1,686 (7.4)	732 (6.6)	817 (8.0)	121 (9.5)	16 (13.3)	377,633 (5.3)
Valacyclovir hydrochloride	1,622 (7.2)	726 (6.6)	780 (7.7)	104 (8.2)	12 (10.0)	389,058 (5.4)
Levofloxacin	1,615 (7.1)	649 (5.9)	809 (7.9)	140 (11.0)	17 (14.2)	229,883 (3.2)

Medicare population

**Prescription (n, %)**	**All ET patients N=10,343**	**0 qualified ET treatments n=6,061**	**1 qualified ET treatment n=3,617**	**2 qualified ET treatments n=597**	**3+ qualified ET treatments n=68**	**All non-ET patients N=429,609**
Atorvastatin calcium	3,410 (33.0)	1,941 (32.0)	1,229 (34.0)	218 (36.5)	22 (32.4)	126,136 (29.4)
Propranolol hydrochloride	2,820 (27.3)	825 (13.6)	1,587 (43.9)	361 (60.5	47 (69.1)	4,21 (71.0)
Amoxicillin	2,596 (25.1)	1,501 (24.8)	924 (25.5)	149 (25.0)	22 (32.4)	93,937 (21.9)
Primidone	2,517 (24.3)	685 (11.3)	1,363 (37.7)	409 (68.5)	60 (88.2)	446 (0.1)
Prednisone	2,476 (23.9)	1,352 (22.3)	937 (25.9)	163 (27.3)	24 (35.3)	79,894 (18.6)
Levothyroxine sodium	2,349 (22.7)	1,322 (21.8)	843 (23.3)	165 (27.6)	19 (27.9)	85,818 (20.0)
Azithromycin	2,339 (22.6)	1,227 (20.2)	932 (25.8)	162 (27.1)	18 (26.5)	79,584 (18.5)
Cephalexin	2,218 (21.4)	1,276 (21.1)	780 (21.6)	146 (24.5)	16 (23.5)	68,273 (15.9)

Amlodipine besylate	2,202 (21.3)	1,242 (20.5)	809 (22.4)	136 (22.8)	15 (22.1)	86,695 (20.2)
Omeprazole	2,186 (21.1)	1,187 (19.6)	833 (23.0)	145 (24.3)	21 (30.9)	65,342 (15.2)
Gabapentin	2,099 (20.3)	627 (10.3)	1,107 (30.6)	315 (52.8)	50 (73.5)	46,407 (10.8)
Acetaminophen/hydrocodone bitartrate	2,021 (19.5)	1,067 (17.6)	787 (21.8)	150 (25.1)	17 (25.0)	62,303 (14.5)
Zoster vaccine recombinant, adjuvanted	1,986 (19.2)	1,263 (20.8)	620 (17.1)	91 (15.2)	12 (17.6)	77,571 (18.1)
Fluticasone propionate	1,949 (18.8)	1,070 (17.7)	728 (20.1)	134 (22.4)	17 (25.0)	60,937 (14.2)
Albuterol sulfate	1,936 (18.7)	1,008 (16.6)	768 (21.2)	149 (25.0)	11 (16.2)	59,844 (13.9)
Ciprofloxacin hydrochloride	1,823 (17.6)	971 (16.0)	710 (19.6)	129 (21.6)	13 (19.1)	54,000 (12.6)
Lisinopril	1,795 (17.4)	1,032 (17.0)	622 (17.2)	122 (20.4)	19 (27.9)	71,860 (16.7)
Metoprolol succinate	1,742 (16.8)	1,147 (18.9)	529 (14.6)	60 (10.1)	6 (8.8)	66,136 (15.4)
Furosemide	1,670 (16.1)	874 (14.4)	638 (17.6)	144 (24.1)	14 (20.6)	48,009 (11.2)
Losartan potassium	1,667 (16.1)	979 (16.2)	592 (16.4)	83 (13.9)	13 (19.1)	65,254 (15.2)
Amoxicillin/clavulanate potassium	1,660 (16.0)	895 (14.8)	631 (17.4)	125 (20.9)	9 (13.2)	55,862 (13.0)
Doxycycline hyclate	1,556 (15.0)	838 (13.8)	586 (16.2)	119 (19.9)	13 (19.1)	47,696 (11.1)
Tramadol hydrochloride	1,552 (15.0)	782 (12.9)	634 (17.5)	119 (19.9)	17 (25.0)	43,865 (10.2)
Tamsulosin hydrochloride	1,524 (14.7)	838 (13.8)	583 (16.1)	95 (15.9)	8 (11.8)	44,873 (10.4)
Methylprednisolone	1,443 (14.0)	755 (12.5)	579 (16.0)	101 (16.9)	8 (11.8)	48,431 (11.3)
Metformin hydrochloride	1,436 (13.9)	755 (12.5)	563 (15.6)	105 (17.6)	13 (19.1)	58,171 (13.5)
Triamcinolone acetonide	1,394 (13.5)	787 (13.0)	504 (13.9)	89 (14.9)	14 (20.6)	46,290 (10.8)
Simvastatin	1,390 (13.4)	780 (12.9)	499 (13.8)	100 (16.8)	11 (16.2)	52,185 (12.1)
Pantoprazole sodium	1,383 (13.4)	745 (12.3)	540 (14.9)	92 (15.4)	6 (8.8)	38,678 (9.0)
Sulfamethoxazole/trimethoprim	1,364 (13.2)	719 (11.9)	543 (15.0)	96 (16.1)	6 (8.8)	42,097 (9.8)
Rosuvastatin calcium	1,175 (11.4)	614 (10.1)	461 (12.7)	88 (14.7)	12 (17.6)	43,953 (10.2)
Hydrochlorothiazide	1,133 (11.0)	627 (10.3)	425 (11.8)	73 (12.2)	8 (11.8)	50,066 (11.7)
Potassium chloride	1,107 (10.7)	595 (9.8)	422 (11.7)	82 (13.7)	8 (11.8)	33,788 (7.9)
Diclofenac sodium	1,104 (10.7)	552 (9.1)	459 (12.7)	77 (12.90	16 (23.5)	34,227 (8.0)
Nitrofurantoin monohydrate/nitrofurantoin, macro	1,036 (10.0)	591 (9.8)	377 (10.4)	62 (10.4)	6 (8.8)	29,971 (7.0)
Mupirocin	1,023 (9.9)	536 (8.8)	412 (11.4)	68 (11.4)	7 (10.3)	30,491 (7.1)
Meloxicam	1,019 (9.9)	551 (9.1)	405 (11.2)	56 (9.4)	7 (10.3)	34,902 (8.1)
Metoprolol tartrate	1,010 (9.8)	648 (10.7)	316 (8.7)	43 (7.2)	3 (4.4)	36,913 (8.6)
Sertraline hydrochloride	1,001 (9.7)	546 (9.0)	383 (10.6)	62 (10.4)	10 (14.7)	22,094 (5.1)
Apixaban	985 (9.5)	559 (9.2)	365 (10.1)	51 (8.5)	10 (14.7)	29,144 (6.8)

Alprazolam	984 (9.5)	446 (7.4)	454 (12.6)	76 (12.7)	8 (11.8)	24,645 (5.7)
Carbidopa/levodopa	982 (9.5)	575 (9.5)	337 (9.3)	66 (11.1)	4 (5.9)	4,876 (1.1)
Levofloxacin	981 (9.5)	465 (7.7)	407 (11.3)	93 (15.6)	16 (23.5)	26,329 (6.1)
Benzonatate	938 (9.1)	365 (6.0)	464 (12.8)	98 (16.4)	11 (16.2)	29,279 (6.8)
Acetaminophen/oxycodone hydrochloride	915 (8.8)	411 (6.8)	401 (11.1)	91 (15.2)	12 (17.6)	24,961 (5.8)
Clopidogrel hydrogen sulfate	907 (8.8)	540 (8.9)	300 (8.3)	64 (10.7)	3 (4.4)	27,807 (6.5)
Escitalopram oxalate	875 (8.5)	469 (7.7)	349 (9.6)	53 (8.9)	4 (5.9)	18,792 (4.4)
Prednisolone acetate	870 (8.4)	497 (8.2)	316 (8.7)	53 (8.9)	4 (5.9)	31,089 (7.2)
Clindamycin hydrochloride	848 (8.2)	464 (7.7)	312 (8.6)	66 (11.1)	6 (8.8)	28,534 (6.6)
Montelukast sodium	836 (8.1)	451 (7.4)	309 (8.5)	68 (11.4)	8 (11.8)	25,583 (6.0)

Key: ET – essential tremor.
