# Supplementary material for: Clinical and Economic Correlates of Pharmacotherapy in Patients with Essential Tremor
Source: Tremor Other Hyperkinet Mov (N Y). 2024 Dec 17;14:60. doi: 10.5334/tohm.973 (PMC11661014; doi:10.5334/tohm.973)
Supplement: Supplementary Table 2. — ET-related healthcare resource utilization and costs. [file tohm-14-1-973-s2.pdf]

S Table 2. ET-related healthcare resource utilization and costs

**Commercial population**					
	**All ET patients N=22,641**	**0 qualified ET treatments n=11,052**	**1 qualified ET treatment n=10,195**	**2 qualified ET treatments n=1,274**	**3+ qualified ET treatments n=120**
Inpatient admission, n (%)	77 (0.3)	21 (0.2)	36 (0.4)	19 (1.5)	1 (0.8)
No., mean (SD)	1.2 (0.4)	1.1 (0.3)	1.1 (0.4)	1.3 (0.5)	1.0 (0.0)
LOS, days, mean (SD)	1.0 (1.2)	1.4 (0.8)	1.7 (1.4)	2.0 (1.1)	1.0 (0.0)
Cost, mean (SD; median)	$57,254 (40,604; 48,609)	$53,770 (36,800; 52,463)	$55,949 (44,064; 45,951)	$65,824 (38,532; 52,418)	$14,575 (0; 14,575)
ER visit, n (%)	423 (1.9)	153 (1.4)	225 (2.2)	43 (3.4)	2 (1.7)
No., mean (SD)	1.5 (0.9)	1.4 (0.8)	1.6 (0.9)	1.7 (1.1)	2.0 (1.4)
Cost, mean (SD; median)	$3,679 (5,380; 1,746)	$3,364 (5,820; 1,407)	$3,827 (4,980; 1,967)	$4,121 (5,923; 1,858)	$1,564 (605; 1,564)
Pharmacy costs, mean (SD; median)	$474 (1,846; 117)	$25 (87; 8)	$448 (1,743; 132)	$1,098 (2,873; 413)	$2,029 (4,409; 812)
Total costs, mean (SD; median)	$2,504 (16,360; 260)	$1,873 (17,185; 207)	$2,799 (13,610; 438)	$5,508 (26,209; 438)	$3,704 (11,910; 472)
**Medicare population**					
	**All ET patients N=10,343**	**0 qualified ET treatments n=6,061**	**1 qualified ET treatment n=3,617**	**2 qualified ET treatments n=597**	**3+ qualified ET treatments n=68**
Inpatient admission (n, %)	55 (0.5)	25 (0.4)	15 (0.4)	12 (2.0)	3 (4.4)
No., mean (SD)	1.1 (0.2)	1.1 (0.3)	1.0 (0.0)	1.0 (0.0)	1.0 (0.0)
LOS, days, mean (SD)	1.8 (1.4)	2.0 (1.6)	2.2 (1.7)	1.3 (0.5)	1.3 (0.6)
Cost, mean (SD; median)	$33,567 (38,166; 21,054)	$24,836 (16,606; 19,853)	$26,044 (27,307; 20,889)	$61,743 (65,128; 36,712)	$31,240 (36,363; 19,471)
ED visit, n (%)	378 (3.7)	240 (4.0)	108 (3.0)	30 (5.0)	0 (0.0)
No., mean (SD)	1.9 (1.3)	2.0 (1.5)	1.6 (0.9)	1.9 (1.5)	0.0 (0.0)
Cost, mean (SD; median)	$2,407 (5,521; 1,461)	$2,021 (3,068; 1,600)	$3,364 (9,136; 1,583)	$2,051 (2,431; 1,145)	$0 (0; 0)
Pharmacy costs, mean (SD; median)	$354 (1,152; 117)	$177 (320; 40)	$351 (1,253; 125)	$742 (1,485; 402)	$1,583 (2,850; 901)

Total costs, mean (SD; median)	$3,230 (17,371; 333)	$2,510 (11,840; 272)	$3,637 (18,196; 420)	$7,415 (41,038; 623)	$9,009 (28,340; 1.094)

Key: ED – emergency department; ET – essential tremor; SD – standard deviation.
